# Supplementary material for: IL-7 receptor blockade blunts antigen-specific memory T cell responses and chronic inflammation in primates
Source: Nat Commun. 2018 Oct 26;9:4483. doi: 10.1038/s41467-018-06804-y (PMC6203796; doi:10.1038/s41467-018-06804-y)
Supplement: Supplementary file 1 — Supplementary Information [file 41467_2018_6804_MOESM1_ESM.pdf]

<u>Supplementary figures and tables</u>

IL-7 receptor blockade blunts antigen-specific memory T cell responses and chronic inflammation in primates

by Belarif et al.

Supplementary Figure 1: *In vitro* functional characterization of anti-IL7Rα mAbs.

(**A**) *Left:* Representative phosphorylated STAT5 (pSTAT5) staining by flow cytometry of unstimulated baboon PBMCs or incubated 15min with 0.1ng/ml of human IL-7 ± 10µg/ml of anti-IL7Rα mAbs. *Right:* IL-7-induced pSTAT5 inhibition with increasing concentration of site-1/2b IgG4 mAb (blue), site-1/2b IgG1 mAb (red), site-1 IgG4 (green) mAbs, or Ig control (grey). (**B**) TSLP-induced CCL17 secretion (normalized to control condition without mAb) by CD1c^{+} dendritic cells with increasing concentration of anti-IL7Rα mAbs (same colors as in (A)) or anti-TSLPR mAb (black). (**C**) *Left:* Specific cytotoxicity (%) induced by anti-IL7Rα mAbs (same colors as in (A)) on transfected IL-7Rα^{+} Ba/F3 cell line cultured 4 hours with NK cells (ratio 1:10). *Right:* Specific cytotoxicity (%) induced with increasing concentration of site-1/2b IgG1 on human T cell leukemia expressing high level of CD127 (DND-41 cell line, solid red line) or low level of CD127 (Jurkat cell line, dotted red line) cultured 4 hours with NK cells (ratio 1:10). Data are mean ± SEM.

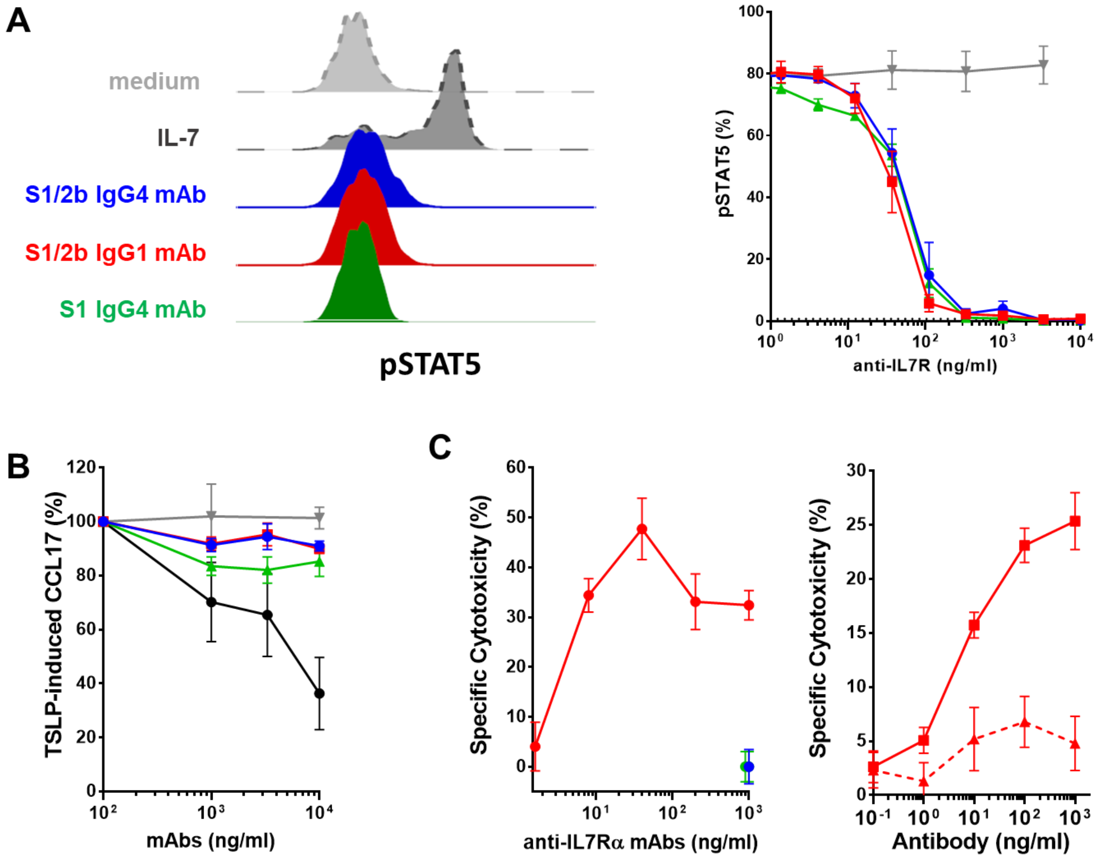

Supplementary Figure 2: Pharmacological evaluation following intravenous administration of anti-IL7Rα mAbs in baboons.

(**A**) Total CD127 expression represented by the Mean Fluorescence Intensity (MFI) measured by flow cytometry on peripheral blood CD3$^+$ baboon T lymphocytes after a single intravenous injection of 10mg/kg of the site-1/2b IgG1 mAb (red, n= 3), site-1/2b IgG4 mAb (blue, n = 3) or site-1 IgG4 mAb (green, n = 3). Dotted line represents the mean basal level of expression before administration of mAbs. (**B**) Serum concentration of anti-IL7Rα mAbs after injection in same animals as in (A). (**C**) CD127 receptor occupancy determined by flow cytometry on peripheral CD3$^+$ T lymphocytes purified from the blood (*left*) or lymph nodes (*right*) of the same animals as in (A). Data are mean ± SEM.

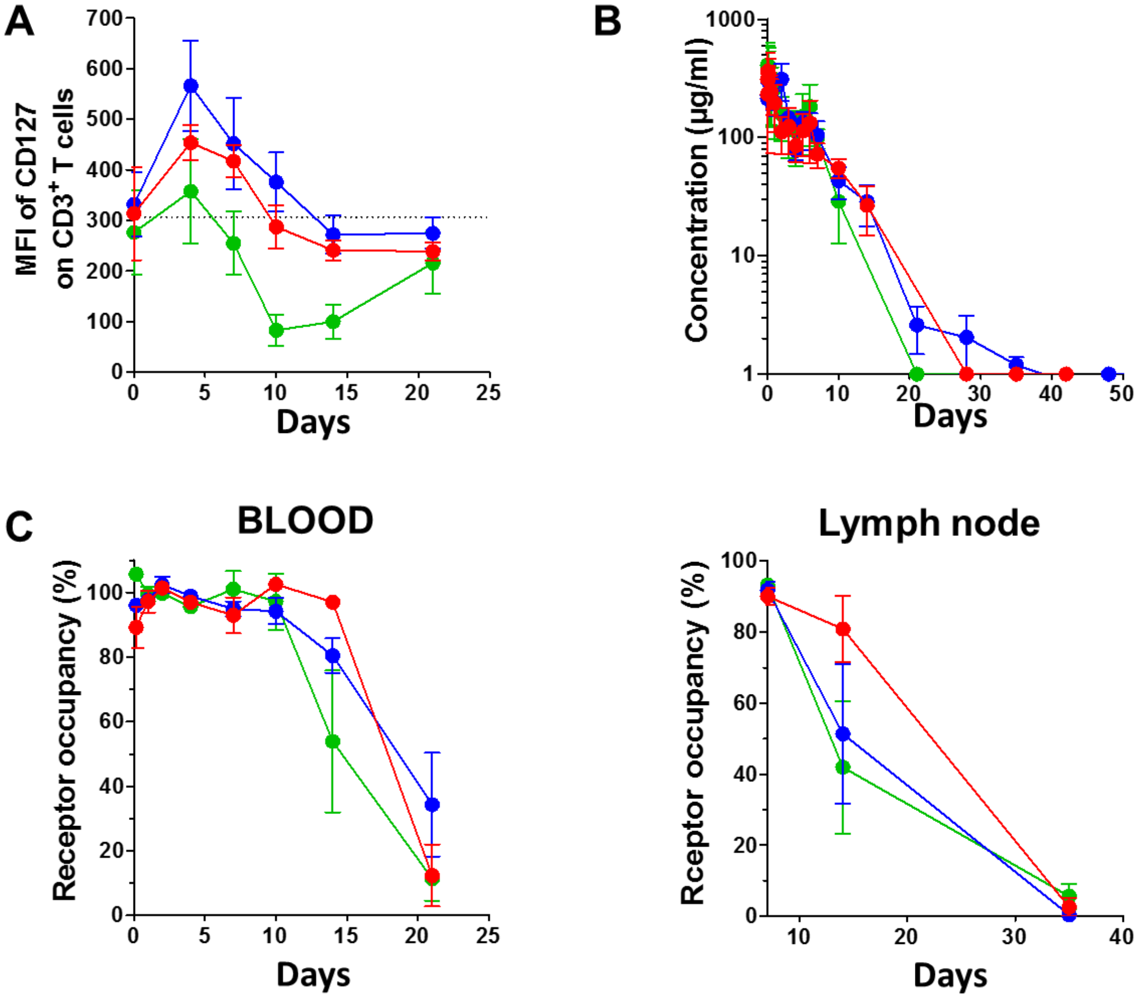

Supplementary Figure 3: Sheep red blood cell (SRBC) immunization.

(**A**) Anti-SRBC IgG fold-change serum titers in baboons treated with a single intravenous injection of 10mg/kg of the site-1/2b IgG1 mAb (red, n= 3), site-1/2b IgG4 mAb (blue, n = 3) or site-1 IgG4 mAb (green, n = 3), and challenged 24 hours later with intravenous injection of 1.5ml/kg of SRBC at 10%. Historical placebo-treated animals (black, n= 3) following the same protocol were used as controls [50]. (**B**) Same as in (A) after secondary SRBC challenge performed 20 weeks following anti-IL7Rα mAb administration. Data are mean ± SEM. * p<0.05 one-way ANOVA and Dunn's test comparison between the site1/2b IgG4 mAb and control group at 2 weeks post-SRBC challenge.

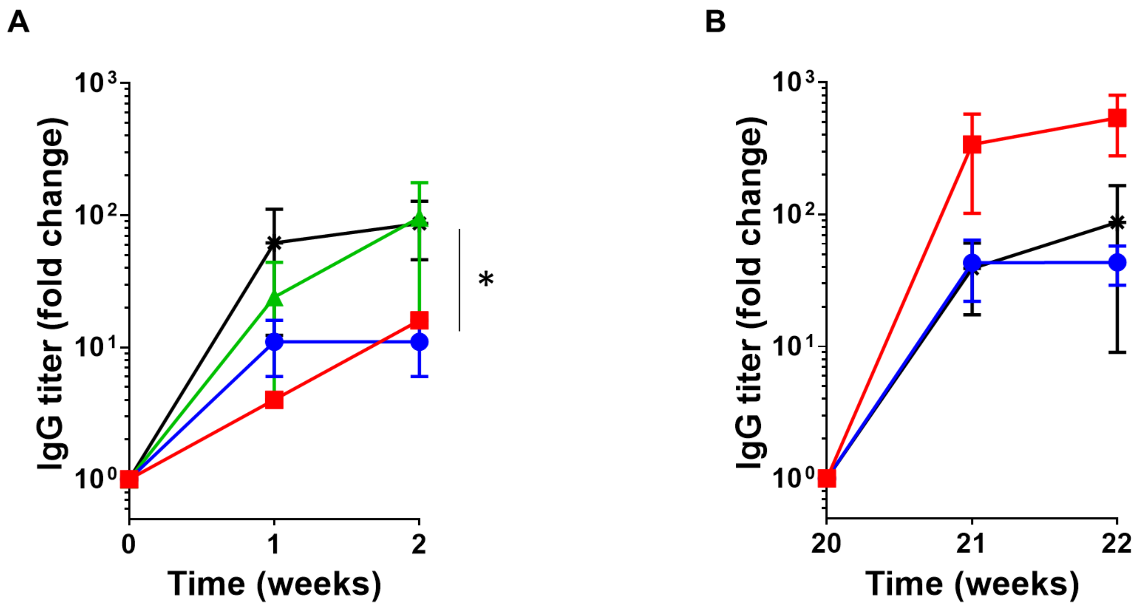

Supplementary Figure 4: Anti-IL-7Rα mAbs in primates do not induce modification of peripheral blood cells.

Peripheral blood immune cell enumeration and T cell subset frequency determined by flow cytometry for baboons treated with a single intravenous injection of 10mg/kg of the site-1/2b IgG1 mAb (red, n= 3), site-1/2b IgG4 mAb (blue, n = 3) or site-1 IgG4 mAb (green, n = 3). T cell sub-populations were defined using the following gating strategy $CD3^+$ $CD4^+$ or $CD8^+$ cells for Tem: effector memory T cells ($CD95^+$ $CD28^-$), Tcm: central memory T cells ($CD95^+$ $CD28^+$), Tn: naïve T cells ($CD95^-$ $CD28^+$), Tregs: regulatory T cells (CD4+ $CD25^{high}$ $Foxp3^+$) as previously described [76]. Data are mean ± SEM of the indicated population.

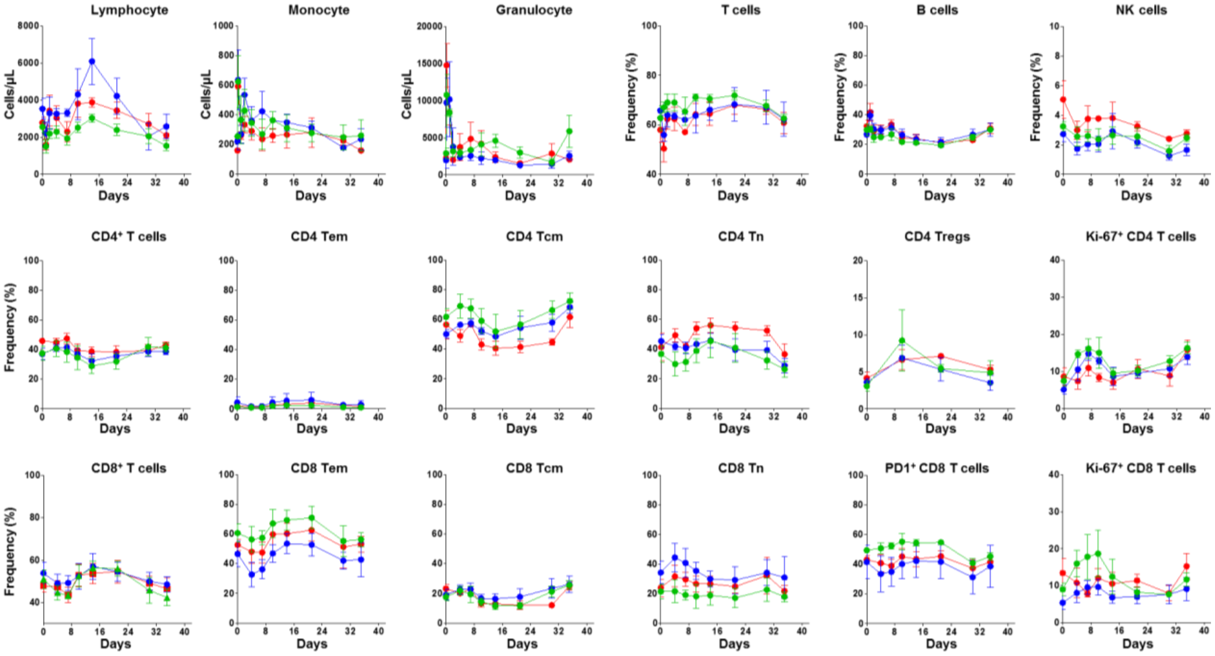

Supplementary Figure 5: Anti-IL7Rα mAb administration does not induce significant peripheral cytokine release.

Serum concentration (pg/mL) of indicated cytokines (IL-2, IL-4, IL-5, IL-6, TNFα and INFγ) in baboons after a single intravenous injection of 10mg/kg of the site-1/2b IgG1 mAb (red, n= 3), site-1/2b IgG4 mAb (blue, n = 3) or site-1 IgG4 mAb (green, n = 3). Data are mean ± SEM.

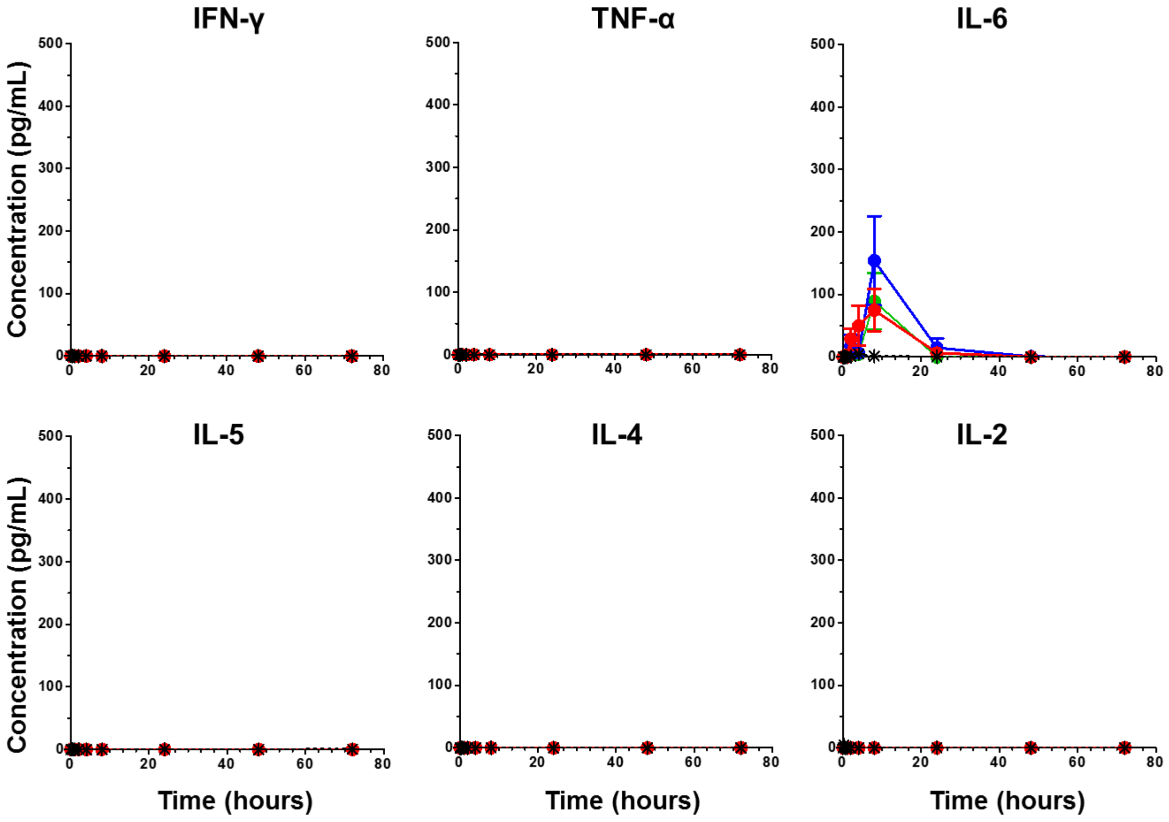

Supplementary Figure 6: Comparison of anti-IL-7Rα site-1 and site1/2b mAbs agonist signals on human cell by western blot.

Raw data of the representative phospho-STAT5, phospho-PI3k-p55, phospho-ERK1/2 and GAPDH western blot of one out of seven representative human donor cells illustrated in Figure 3C. PBMCs were pretreated with 10µg/mL of one anti-IL-7Rα mAb and then incubated for 10 min at 37°C with or without 5ng/ml of human IL-7.

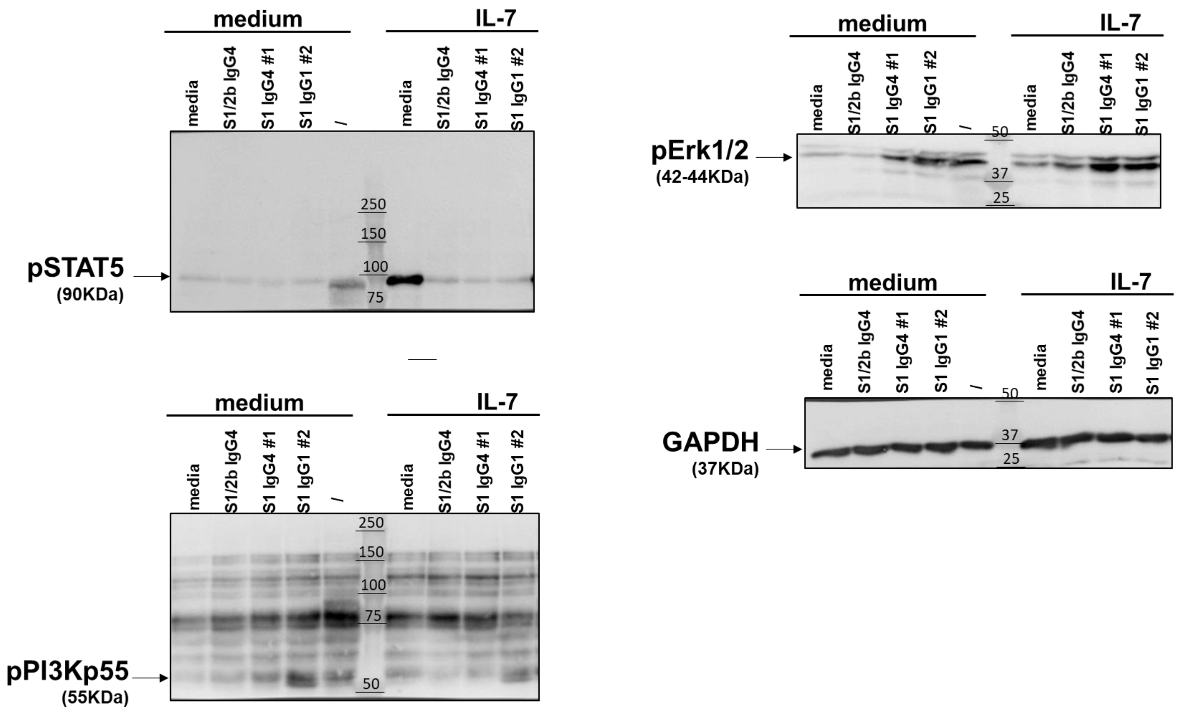

Supplementary Figure 7: Comparison of anti-IL-7Rα site-1 and site1/2b mAbs agonist signals on human and baboon PBMC.

Phospho-STAT5, phospho-PI3k-p55, phospho-ERK1/2 and GAPDH western blot on human (**A**) or baboon cells (**B**). PBMCs were pretreated with 10µg/mL of anti-IL-7Rα mAbs (Site 1/2b IgG4, Site 1/2b IgG1, Site 1 IgG4 #1, Site 1 IgG1 #1, Site 1 IgG1 #2 and Site 1 IgG4 #3) or human isotype control (Iso hIgG4 and Iso hIgG1) and then incubated for 10 min at 37°C before protein lysates analysis.

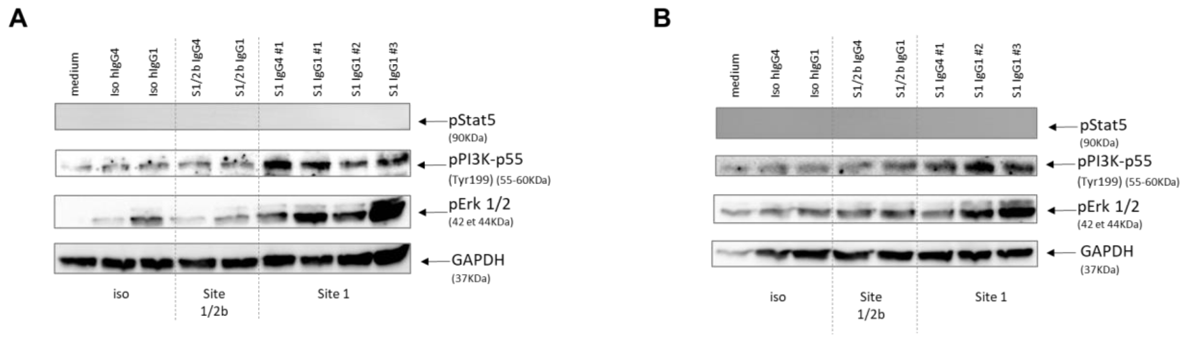

Supplementary Figure 8: Gene Ontology (GO) identified in the signature for each anti-IL-7Rα mAb (blue: site-1/2b IgG4, red: site-1 IgG4#1, green: site-1 IgG1#2) versus control. Circle size is proportional to the number of genes for each category.

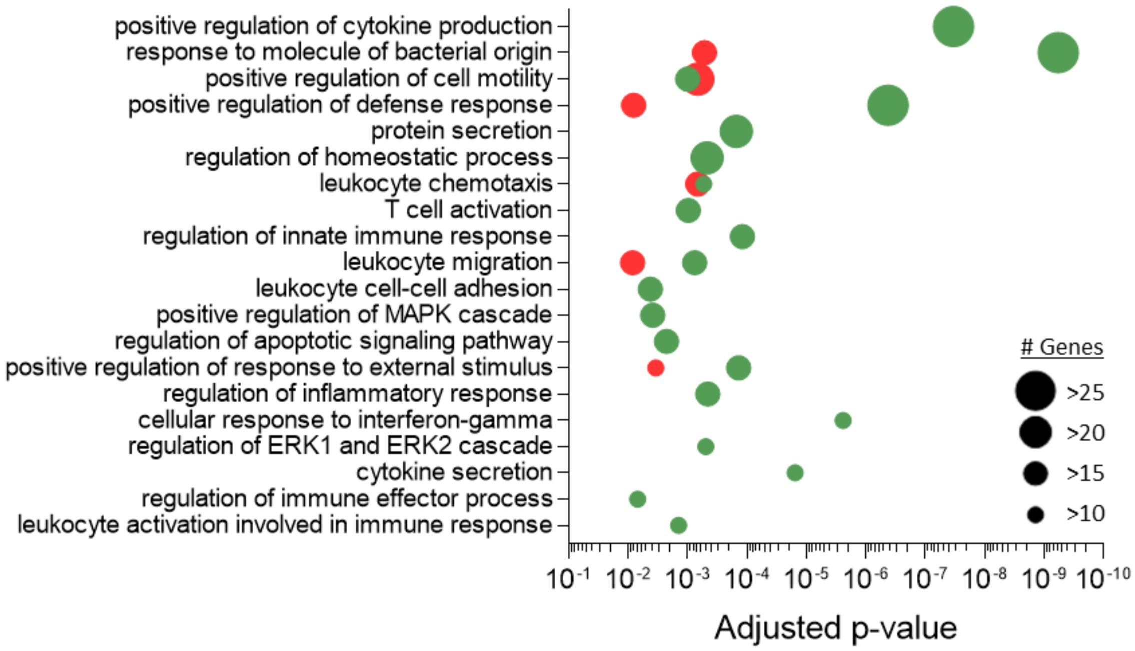

Supplementary Figure 9: GoMiner gene ontology enrichment of the three clusters identified for the 93 most differentially expressed genes (FDR 5%, FC > 2) between IL-7 stimulation and control medium condition.

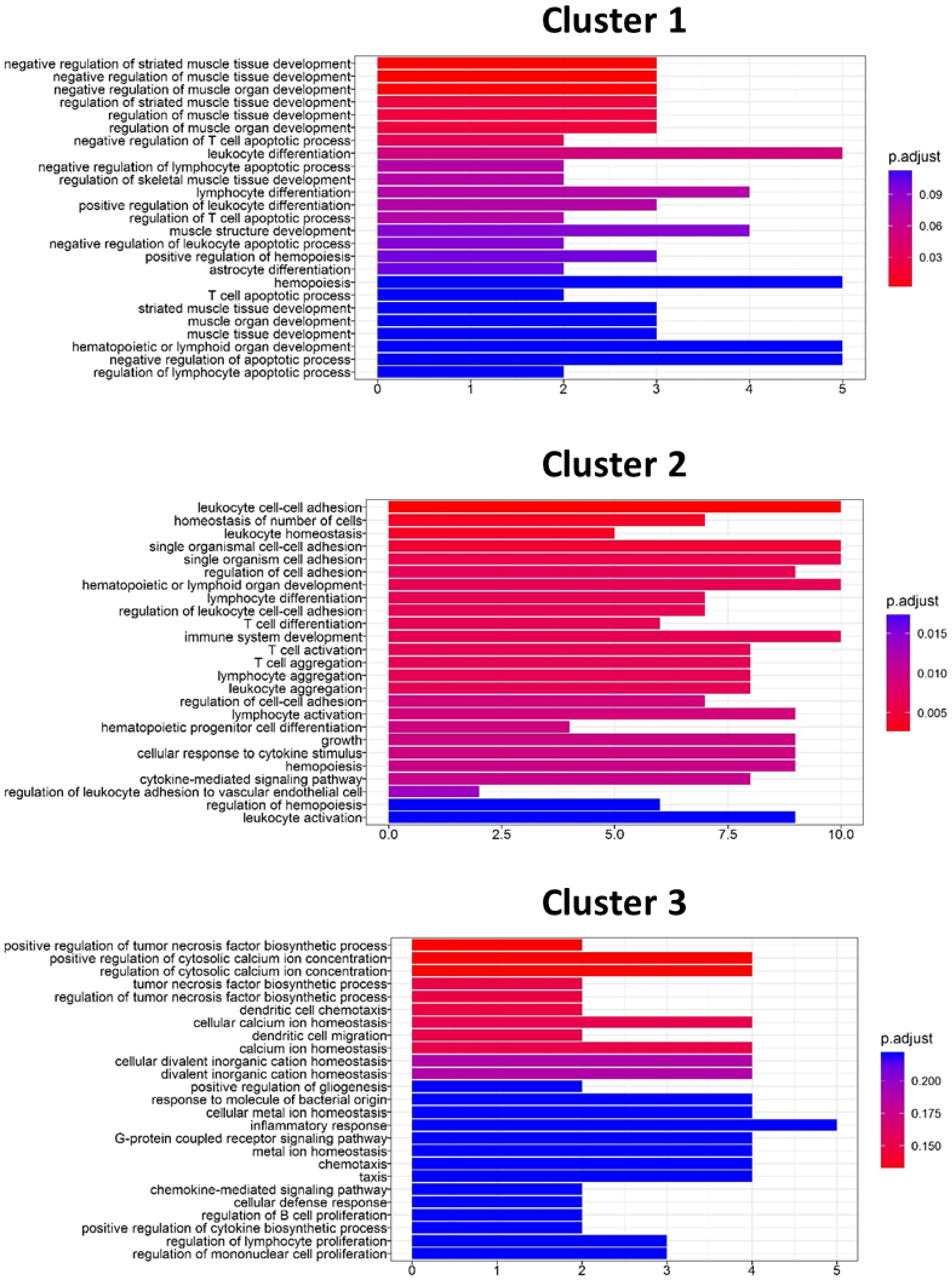

Supplementary Figure 10: IKZF4 gene expression.

IKZF4 mRNA expression confirmed by RT-qPCR on human PBMC cultured for 3.5 hours with (empty round) or without (full symbols) 5 ng/ml of IL-7 and 10 µg/ml of anti-human IL-7Rα mAbs (blue: site-1/2b IgG4, red: site-1 IgG4#1, green: site-1 IgG1#2). Data were normalized to basal expression in the absence of IL-7 and mAbs. * p<0.05 between indicated groups.

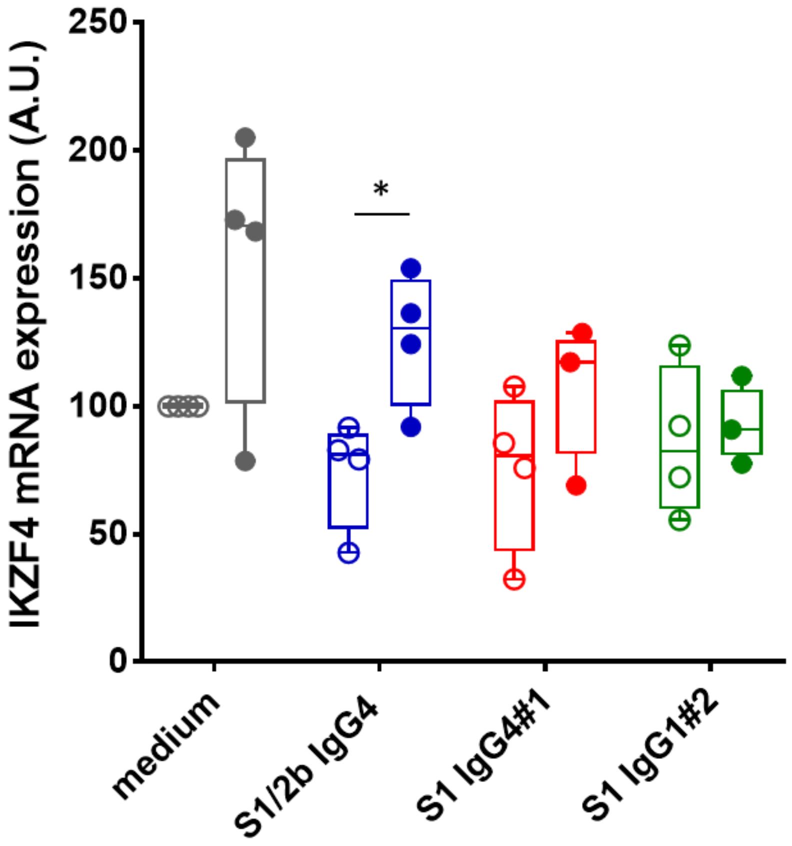

Supplementary Figure 11: Erythema and Elispot response in the non-responder animal. Cutaneous erythema response (black histograms, *left-axis*) at indicated time-points, represented with area under the curve (AUC) of daily erythema diameters, after tuberculin intradermal injection in the non-responder baboon (1 out of 10) treated with a single intravenous injection of 10mg/Kg of the humanized site-1/2b IgG4 mAb. IFN-γ-secreting cell frequencies (white histograms, *right-axis*) in PBMCs of the same animal after *ex-vivo* tuberculin restimulation at the indicated time-point.

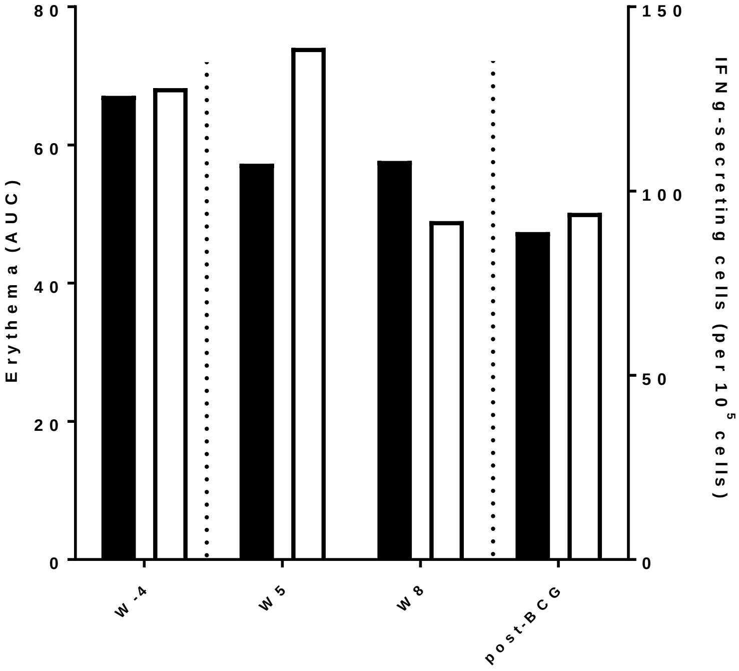

Supplementary Figure 12: <u>CD25 and C69 expression on baboon CD3$^+$ T cells population before and after stimulation.</u>

Representative expression of CD25 and CD69 analyzed by flow cytometry on peripheral blood T-cell (CD3+) at the indicated time-point after treatment with a single intravenous injection of 10 mg/kg of a humanized site-1/2b IgG4 mAb. CD25 and CD69 expression was analyzed on T cells in unstimulated or after 48 hours of anti-CD3 polyclonal stimulation.

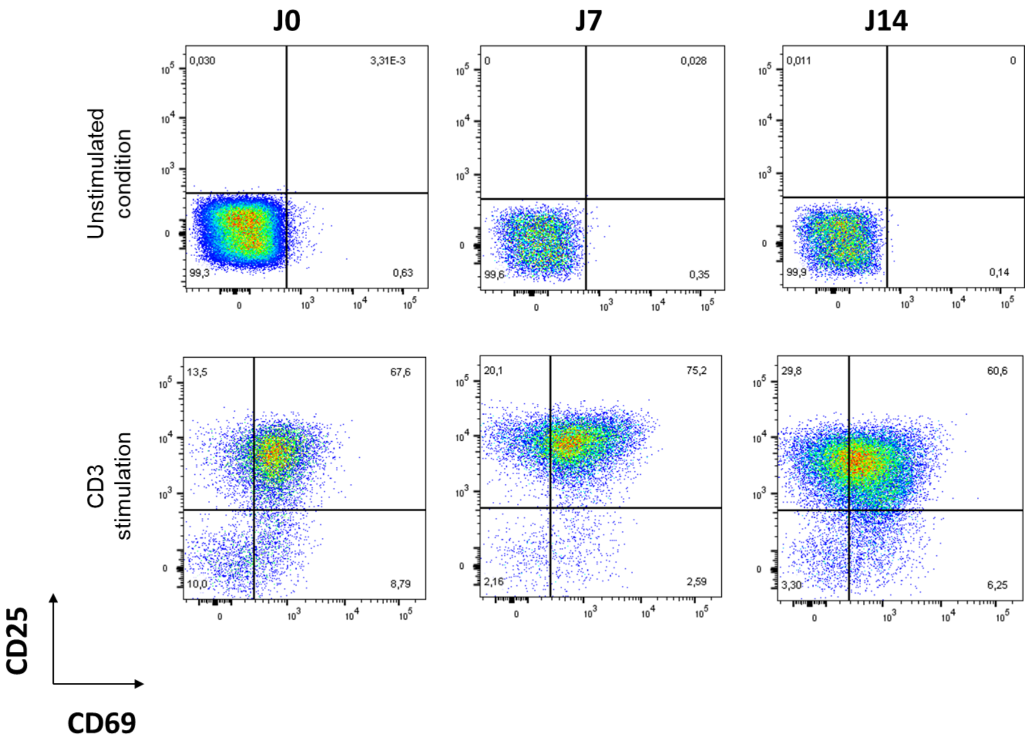

Supplementary Table 1: List of genes significantly (FDR 5%) and differentially (fold-change >1.5) expressed after incubation of human PBMCs (n=7) with anti-IL7Rα mAbs compared to unstimulated cells.

Gene	Site 1/2b IgG4 logFC /Unstim	Site 1/2b IgG4 adj.P.Val	Site 1/2b IgG4 Differential	Site 1 IgG4 #1 logFC /Unstim	Site 1 IgG4 #1 adj.P.Val	Site 1 IgG4 #1 Differential	Site 1 IgG1 #2 logFC /Unstim	Site 1 IgG1 #2 adj.P.Val	Site 1 IgG1 #2 Differential
ACADVL	0,85	0,040	*	1,15	0,001	*	0,87	0,011	*
AHR	0,86	0,029	*	1,16	0,000	*	1,21	0,000	*
AKIRIN1	0,75	0,028	*	0,88	0,002	*	0,78	0,006	*
ALDH16A1	1,23	0,021	*	1,45	0,001	*	1,32	0,003	*
ALDH5A1	0,72	0,044	*	0,93	0,002	*	1,05	0,001	*
APPBP2	0,81	0,001	*	0,90	0,000	*	0,81	0,000	*
ARHGEF1	0,85	0,001	*	1,05	0,000	*	0,88	0,000	*
B3GNT2	1,03	0,034	*	1,23	0,003	*	1,15	0,005	*
C17orf59	1,07	0,039	*	1,49	0,001	*	1,27	0,004	*
CCDC117	0,77	0,024	*	0,88	0,002	*	0,80	0,005	*
CCNI	0,73	0,004	*	0,98	0,000	*	0,78	0,001	*
CDK17	0,98	0,003	*	1,09	0,000	*	0,94	0,001	*
COTL1	1,67	0,000	*	1,80	0,000	*	1,35	0,000	*
CYP1B1	-0,66	0,029	*	-0,75	0,003	*	-0,69	0,006	*
DDI2	1,17	0,011	*	1,45	0,000	*	1,24	0,002	*
DEF6	1,03	0,030	*	1,39	0,001	*	1,09	0,006	*
DNLZ	1,17	0,029	*	1,19	0,008	*	1,22	0,006	*
DUSP2	1,73	0,002	*	2,18	0,000	*	2,30	0,000	*
ENG	-0,73	0,004	*	-0,61	0,005	*	-0,67	0,002	*
EXOC8	0,69	0,043	*	0,62	0,033	*	0,63	0,026	*
FAM160B1	0,83	0,030	*	1,26	0,000	*	0,72	0,024	*
FEM1B	0,99	0,005	*	1,09	0,000	*	0,87	0,005	*
GCFC2	1,00	0,011	*	0,87	0,011	*	0,83	0,013	*
GNA15	0,99	0,011	*	1,14	0,001	*	1,25	0,000	*
GOPC	0,83	0,015	*	0,64	0,029	*	0,69	0,015	*
GRSF1	0,65	0,015	*	0,81	0,000	*	0,69	0,002	*
HIPK3	0,67	0,025	*	0,79	0,002	*	0,69	0,006	*
HMHA1	0,88	0,004	*	1,05	0,000	*	0,89	0,001	*
HSBP1L1	1,04	0,029	*	0,97	0,017	*	0,86	0,030	*
JUNB	0,91	0,025	*	1,25	0,000	*	1,42	0,000	*
KLC2	1,08	0,036	*	1,18	0,007	*	1,34	0,002	*
LYSMD2	0,78	0,016	*	0,72	0,010	*	0,70	0,009	*
LYZ	-0,60	0,031	*	-0,86	0,000	*	-0,76	0,001	*
MS4A7	-0,80	0,001	*	-0,98	0,000	*	-0,59	0,006	*
MTA2	0,84	0,038	*	1,18	0,001	*	1,10	0,002	*
NCOA5	0,65	0,018	*	0,84	0,000	*	0,89	0,000	*
NSUN2	0,63	0,029	*	0,74	0,002	*	0,75	0,002	*
PITHD1	0,81	0,040	*	1,03	0,002	*	0,81	0,013	*
PLEKHF2	0,72	0,004	*	0,83	0,000	*	0,77	0,001	*
POLRMT	1,09	0,001	*	1,30	0,000	*	1,06	0,000	*
PPP2CA	0,75	0,025	*	0,87	0,002	*	0,75	0,007	*
PREB	0,88	0,018	*	0,78	0,014	*	0,92	0,004	*
PRKCH	0,70	0,011	*	0,88	0,000	*	0,78	0,001	*
PSMD3	1,01	0,035	*	1,58	0,000	*	1,37	0,001	*
PYGO2	1,08	0,003	*	1,07	0,001	*	0,74	0,016	*
RASSF5	0,63	0,004	*	0,67	0,000	*	0,60	0,002	*
RBL2	0,71	0,011	*	0,85	0,000	*	0,59	0,012	*
RRP1	0,69	0,019	*	0,62	0,013	*	0,80	0,002	*
SMCHD1	0,71	0,001	*	1,00	0,000	*	0,85	0,000	*
SREBF2	0,83	0,009	*	1,13	0,000	*	0,98	0,000	*
TAF10	1,87	0,001	*	1,57	0,001	*	1,28	0,006	*
TAF4B	0,75	0,030	*	1,12	0,000	*	1,29	0,000	*
TMX4	1,04	0,009	*	1,13	0,001	*	0,89	0,009	*
TPGS1	2,55	0,008	*	2,95	0,000	*	2,06	0,011	*
TRAM1	0,71	0,001	*	0,73	0,000	*	0,59	0,002	*
TRPC4AP	0,98	0,005	*	1,21	0,000	*	1,06	0,001	*
TTC13	0,76	0,034	*	0,90	0,003	*	0,68	0,022	*
UNC119	1,02	0,036	*	1,43	0,001	*	1,26	0,002	*
USP9X	0,90	0,000	*	0,92	0,000	*	0,85	0,000	*
VPS4A	0,74	0,042	*	0,95	0,002	*	0,75	0,012	*
ZNF800	0,74	0,013	*	1,01	0,000	*	0,84	0,001	*
ACTN4	0,55	0,005		0,60	0,000	*	0,65	0,000	*
C15orf48	-0,63	0,029	*	-0,87	0,000	*	-0,51	0,035	
C3AR1	-0,56	0,011		-0,75	0,000	*	-0,64	0,001	*
CD247	0,80	0,004	*	0,87	0,000	*	0,50	0,038	
FAM50A	0,66	0,028	*	0,93	0,000	*	0,54	0,031	
HCK	-0,65	0,009	*	-0,83	0,000	*	-0,46	0,030	
IL6ST	0,43	0,034		0,60	0,000	*	0,77	0,000	*
JAK1	0,71	0,001	*	0,80	0,000	*	0,58	0,002	
KYNU	-0,60	0,018	*	-0,94	0,000	*	-0,56	0,007	
NCF2	-0,57	0,029		-0,71	0,001	*	-0,70	0,001	*
PARP10	0,58	0,037		0,75	0,002	*	0,73	0,002	*
RAD54L2	0,61	0,037	*	0,66	0,008	*	0,53	0,033	
SMG5	0,54	0,033		0,80	0,000	*	0,75	0,001	*
TOR3A	0,73	0,028	*	0,62	0,028	*	0,57	0,040	
ADM	-0,88	0,005	*	-1,01	0,000	*	-0,02	0,967	
CES1	-0,77	0,028	*	-0,78	0,008	*	-0,45	0,145	
SLC31A2	-0,79	0,004	*	-0,60	0,012	*	-0,17	0,577	
SMPDL3A	-1,11	0,009	*	-1,12	0,002	*	-0,56	0,149	
TREM1	-0,72	0,009	*	-0,67	0,005	*	-0,28	0,287	
VNN1	-0,97	0,030	*	-1,17	0,002	*	-0,35	0,423	
FLT1	-1,01	0,028	*	-0,66	0,101		-0,80	0,039	*
ABCG1	0,39	0,339		0,70	0,028	*	0,62	0,047	*
ANKRD30BL	0,88	0,342		1,42	0,049	*	1,73	0,012	*
C16orf58	0,42	0,219		0,63	0,019	*	0,59	0,026	*
C6orf120	0,31	0,536		1,03	0,003	*	0,84	0,013	*
CA2	-0,33	0,450		-0,70	0,033	*	-0,73	0,022	*
CCNL2	0,51	0,178		0,75	0,012	*	0,59	0,049	*
CD14	-0,64	0,070		-0,98	0,001	*	-0,89	0,002	*
CDK16	0,64	0,118		0,96	0,004	*	0,80	0,014	*
CHTF18	0,67	0,174		0,87	0,030	*	1,13	0,004	*
CLPTM1	0,74	0,111		1,06	0,005	*	1,01	0,007	*
CREBZF	0,50	0,116		0,72	0,006	*	0,67	0,008	*
CREG1	0,70	0,095		0,86	0,013	*	0,99	0,004	*
CSF1R	-0,39	0,263		-0,71	0,010	*	-0,92	0,001	*
DAPK3	0,56	0,240		0,80	0,038	*	0,97	0,008	*
DDRGK1	0,91	0,126		1,39	0,004	*	1,53	0,002	*
DOHH	1,40	0,061		1,45	0,020	*	1,69	0,006	*
EMC8	0,61	0,229		1,15	0,004	*	1,04	0,008	*
FAM115C	0,37	0,264		0,61	0,022	*	0,64	0,013	*
FUCA1	-0,35	0,234		-0,59	0,011	*	-0,66	0,004	*
GAPT	-0,48	0,188		-0,67	0,023	*	-0,85	0,003	*
GMIP	0,55	0,076		0,78	0,002	*	0,70	0,005	*
GMPPB	0,82	0,073		0,92	0,016	*	0,95	0,010	*
HCG11	0,70	0,087		1,05	0,002	*	0,80	0,014	*
HEATR3	0,43	0,280		0,74	0,019	*	0,74	0,017	*
HEIH	0,97	0,056		1,05	0,014	*	0,84	0,047	*
HELZ2	0,42	0,287		0,77	0,013	*	0,77	0,010	*
HMG20B	0,54	0,088		0,61	0,019	*	0,59	0,022	*
HNRNPA0	0,58	0,165		0,74	0,028	*	0,90	0,006	*
HPCAL1	0,38	0,344		0,86	0,005	*	0,67	0,028	*
KIAA1919	0,63	0,116		0,81	0,013	*	0,66	0,042	*
KLHDC2	0,54	0,149		0,71	0,019	*	0,62	0,040	*
LTB	0,45	0,248		0,92	0,003	*	0,82	0,007	*
MAF1	0,86	0,161		1,54	0,002	*	1,38	0,005	*
MAFF	0,54	0,249		0,81	0,029	*	1,19	0,001	*
MAP3K11	0,49	0,263		0,77	0,030	*	0,70	0,044	*
MGST1	-1,05	0,089		-1,24	0,015	*	-1,06	0,036	*
MIER2	0,89	0,112		0,93	0,047	*	1,25	0,006	*
MOB2	0,69	0,150		1,05	0,007	*	0,90	0,017	*
PET100	-0,32	0,298		-0,61	0,013	*	-0,61	0,010	*
PLK3	0,20	0,700		0,79	0,021	*	0,83	0,012	*
POLG	0,52	0,086		0,97	0,000	*	0,79	0,002	*
PSMD2	0,41	0,212		0,62	0,018	*	0,61	0,017	*
RAB13	-0,50	0,089		-0,73	0,002	*	-0,62	0,009	*
RASAL3	0,74	0,067		0,92	0,006	*	0,72	0,028	*
RELB	0,22	0,633		0,63	0,046	*	0,69	0,022	*
RGMB	0,58	0,173		0,80	0,020	*	0,74	0,028	*
RNF10	0,56	0,095		0,67	0,016	*	0,66	0,014	*
RNF149	0,60	0,077		0,69	0,016	*	0,69	0,012	*
RPL39L	-1,02	0,125		-1,09	0,049	*	-1,41	0,008	*
RPUSD1	0,76	0,105		0,84	0,031	*	1,00	0,008	*
SCARF1	0,39	0,408		0,77	0,032	*	1,13	0,002	*
SENP6	0,44	0,204		0,69	0,012	*	0,72	0,007	*
SIPA1L3	-0,01	0,992		0,71	0,021	*	0,74	0,013	*
SLC25A22	0,51	0,283		0,75	0,047	*	0,83	0,024	*
SLC38A10	0,24	0,595		0,76	0,014	*	0,66	0,030	*
SLC43A2	0,66	0,068		0,63	0,040	*	0,99	0,001	*
SPATC1L	0,96	0,180		1,17	0,044	*	1,22	0,031	*
SPP1	-0,53	0,102		-0,85	0,001	*	-0,62	0,017	*
SRP68	0,68	0,096		0,77	0,024	*	0,98	0,003	*
TAZ	0,95	0,092		1,23	0,008	*	1,22	0,007	*
TCIRG1	0,79	0,069		1,15	0,001	*	1,15	0,002	*
TNFRSF1B	0,51	0,208		0,72	0,026	*	0,82	0,009	*
TNFRSF4	0,23	0,751		0,97	0,034	*	1,15	0,009	*
TNKS	0,66	0,052		0,78	0,006	*	0,67	0,016	*
TSC22D2	0,47	0,137		0,65	0,011	*	0,61	0,014	*
UBA5	0,91	0,076		1,22	0,004	*	1,17	0,005	*
USP48	0,64	0,145		0,90	0,012	*	0,72	0,044	*
VPS51	0,50	0,145		0,82	0,003	*	0,76	0,005	*
ZFAND5	0,56	0,054		0,71	0,003	*	0,64	0,007	*
ZNF259	0,47	0,242		0,85	0,007	*	0,65	0,038	*
ZNF496	0,51	0,220		0,86	0,008	*	0,83	0,009	*
ZNF696	0,80	0,061		1,09	0,002	*	0,75	0,033	*
ANXA1	-0,43	0,034		-0,49	0,005		-0,63	0,000	*
C12orf75	-0,45	0,041		-0,43	0,022		-0,61	0,001	*
C18orf32	-0,56	0,040		-0,61	0,008	*	-0,48	0,036	
CHMP7	0,50	0,028		0,64	0,001	*	0,55	0,004	
DNAJC3	-0,49	0,004		-0,62	0,000	*	-0,36	0,013	
EVI2B	-0,36	0,046		-0,38	0,013		-0,75	0,000	*
HCST	-0,54	0,034		-0,45	0,042		-0,65	0,003	*
HSBP1	-0,44	0,009		-0,64	0,000	*	-0,46	0,002	
PAPOLA	0,57	0,018		0,72	0,000	*	0,55	0,006	
PPBP	-0,55	0,018		-0,65	0,001	*	-0,50	0,010	
TIMP1	-0,52	0,019		-0,65	0,001	*	-0,57	0,002	
TLR2	-0,42	0,044		-0,60	0,001	*	-0,35	0,048	
UBE2D2	0,49	0,040		0,74	0,000	*	0,49	0,013	
AGTRAP	-0,52	0,040		-0,66	0,002	*	-0,42	0,051	
CXCL16	-0,55	0,034		-0,60	0,006	*	-0,38	0,091	
P2RX4	-0,48	0,034		-0,60	0,002	*	-0,06	0,846	
P2RX7	-0,72	0,018	*	-0,54	0,039		0,32	0,258	
SLAMF7	-0,44	0,011		-0,64	0,000	*	-0,19	0,251	
SLC2A6	-0,59	0,028	*	-0,56	0,013		-0,13	0,648	
SUGP2	0,64	0,005	*	0,50	0,012		0,33	0,114	
TNFAIP6	-0,55	0,029		-0,90	0,000	*	-0,13	0,637	
TNIP3	-0,55	0,028		-0,61	0,004	*	-0,21	0,385	
CYBA	-0,53	0,030		-0,39	0,065		-0,63	0,002	*
EPG5	0,59	0,034	*	0,39	0,112		0,57	0,013	
RNF135	-0,57	0,043		-0,33	0,209		-0,64	0,006	*
ALDH1B1	-0,79	0,036	*	-0,38	0,294		-0,36	0,311	
CKS2	-0,85	0,048	*	-0,61	0,109		-0,59	0,116	
ETFDH	-0,71	0,003	*	-0,36	0,090		-0,40	0,054	
FPR1	-0,87	0,029	*	-0,62	0,076		-0,29	0,467	
GSTT1	-0,94	0,030	*	-0,52	0,195		-0,46	0,254	
NFYB	-0,61	0,025	*	-0,42	0,075		-0,14	0,633	
PREP	0,72	0,030	*	0,48	0,102		0,48	0,092	
TGFBI	-0,99	0,007	*	-0,27	0,477		-0,36	0,310	
TMEM160	-0,75	0,030	*	-0,30	0,373		-0,55	0,062	
TYMP	-0,75	0,040	*	-0,38	0,278		-0,17	0,687	
UBE3D	-0,96	0,046	*	-0,35	0,477		-0,33	0,497	
ZNF619	0,79	0,043	*	0,49	0,170		0,52	0,128	
ABCA1	-0,43	0,068		-0,60	0,002	*	-0,39	0,044	
ACTR6	-0,31	0,332		-0,65	0,007	*	-0,56	0,020	
ANXA5	-0,40	0,089		-0,66	0,001	*	-0,39	0,046	
C16orf70	-0,21	0,548		-0,56	0,021		-0,68	0,005	*
CAND1	0,54	0,102		0,56	0,046		0,88	0,001	*
CAPG	-0,50	0,076		-0,74	0,001	*	-0,48	0,036	
CCDC66	-0,41	0,204		-0,55	0,037		-0,70	0,006	*

COMMD8	-0,33	0,165	-0,59	0,002	*	-0,40	0,034	
CPD	0,52	0,055	0,46	0,043		0,64	0,004	*
CYB5R1	-0,25	0,327	-0,51	0,011		-0,72	0,000	*
DUSP6	-0,45	0,129	-0,61	0,013	*	-0,48	0,049	
ENY2	-0,45	0,067	-0,46	0,026		-0,59	0,004	*
FCER1G	-0,45	0,165	-0,75	0,004	*	-0,51	0,049	
FRMD8	0,52	0,053	0,72	0,001	*	0,49	0,028	
GSR	0,43	0,086	0,71	0,001	*	0,50	0,014	
KIF1B	0,45	0,092	0,59	0,007	*	0,57	0,008	
MEF2D	0,53	0,103	0,60	0,028	*	0,57	0,035	
MPZL1	0,38	0,220	0,58	0,020		0,75	0,002	*
MRPS6	-0,43	0,075	-0,55	0,006		-0,88	0,000	*
NDUFA5	-0,25	0,247	-0,60	0,000	*	-0,36	0,037	
NDUFB7	-0,37	0,213	-0,49	0,043		-0,62	0,007	*
NUP153	0,34	0,228	0,57	0,009		0,62	0,005	*
S100A4	-0,41	0,120	-0,54	0,014		-0,82	0,000	*
S100A8	-0,38	0,266	-0,56	0,043		-0,77	0,004	*
SERPINA1	-0,46	0,067	-0,69	0,001	*	-0,47	0,021	
SLC17A5	-0,33	0,173	-0,51	0,010		-0,72	0,000	*
SLC7A7	-0,47	0,076	-0,68	0,002	*	-0,52	0,016	
SNRK-AS1	0,59	0,079	0,57	0,042		0,59	0,031	*
STAT5A	0,34	0,166	0,66	0,001	*	0,49	0,012	
TBXAS1	-0,27	0,390	-0,49	0,043		-0,64	0,007	*
TFPI2	-0,52	0,079	-0,65	0,008	*	-0,49	0,042	
TMEM184B	0,22	0,545	0,63	0,013	*	0,55	0,027	
TYROBP	-0,35	0,212	-0,56	0,012		-0,84	0,000	*
USMG5	-0,39	0,120	-0,49	0,018		-0,68	0,001	*
USP19	0,48	0,145	0,76	0,005	*	0,54	0,044	
USP38	0,44	0,105	0,49	0,029		0,61	0,006	*
XYLT2	0,27	0,329	0,60	0,005	*	0,57	0,006	
ACTR5	0,56	0,137	0,74	0,018	*	0,53	0,093	
ADAMDEC1	-0,88	0,095	-1,24	0,004	*	-0,63	0,176	
ARHGAP18	-0,55	0,094	-0,63	0,021	*	-0,51	0,060	
AUH	1,01	0,101	1,14	0,026	*	0,67	0,223	
BAG2	0,29	0,548	0,70	0,042	*	0,42	0,254	
BAIAP2	-0,44	0,367	-0,82	0,031	*	-0,32	0,474	
BLOC1S3	-0,54	0,180	-0,71	0,029	*	-0,37	0,290	
C16orf54	0,44	0,128	0,62	0,008	*	0,20	0,477	
C1orf216	0,25	0,669	0,82	0,042	*	0,55	0,193	
C2orf49	-0,47	0,455	-0,97	0,040	*	-0,44	0,407	
C3orf58	0,46	0,182	0,60	0,032	*	0,43	0,144	
CAMK1D	0,47	0,228	0,64	0,040	*	0,48	0,136	
CCL2	-0,33	0,522	-0,86	0,019	*	0,52	0,186	
CCL7	-0,48	0,282	-0,90	0,010	*	0,36	0,355	
CCRL2	-0,42	0,263	-0,76	0,011	*	0,09	0,848	
CD33	-1,08	0,261	-1,61	0,039	*	-1,28	0,103	
CD6	0,12	0,833	0,67	0,042	*	0,51	0,134	
CD68	-0,35	0,184	-0,66	0,002	*	-0,36	0,093	
CDC42EP2	-0,30	0,490	-0,66	0,037	*	0,08	0,864	
CLEC4E	-0,61	0,086	-0,94	0,001	*	-0,53	0,069	
CLEC7A	-0,35	0,521	-0,83	0,034	*	-0,51	0,223	
CPNE8	-0,52	0,422	-1,02	0,036	*	-0,32	0,601	
CSF3	0,03	0,959	-0,63	0,030	*	0,30	0,353	
CTSF	0,32	0,628	1,25	0,006	*	0,88	0,051	
DAB2	-0,62	0,089	-0,67	0,029	*	-0,49	0,119	
DFNA5	-0,63	0,080	-0,89	0,002	*	-0,33	0,311	
ECI1	0,62	0,167	0,78	0,031	*	0,45	0,249	
ELAVL1	0,56	0,120	0,74	0,013	*	0,51	0,091	
ERO1L	-0,52	0,086	-0,85	0,001	*	-0,31	0,252	
EXOSC4	-0,51	0,130	-0,69	0,013	*	-0,23	0,491	
FAM105A	0,50	0,080	0,76	0,001	*	0,44	0,064	
FAM83G	0,39	0,431	0,82	0,029	*	0,71	0,055	
FCAR	-0,32	0,280	-0,71	0,002	*	-0,31	0,211	
FGR	-0,41	0,067	-0,67	0,000	*	-0,27	0,169	
FHL3	0,57	0,180	0,72	0,035	*	0,24	0,572	
FKBP8	0,39	0,166	0,68	0,002	*	0,28	0,250	
FLI1	0,63	0,053	1,03	0,000	*	0,51	0,065	
FPR2	-0,36	0,529	-0,88	0,032	*	0,11	0,867	
G0S2	-0,40	0,231	-0,65	0,015	*	0,02	0,973	
GEMIN2	-0,13	0,855	-0,85	0,041	*	-0,38	0,420	
GGH	-0,45	0,302	-0,73	0,034	*	-0,59	0,091	
GIMAP1	0,39	0,256	0,69	0,012	*	0,47	0,097	
GIMAP1-GIMAP5	1,89	0,075	1,77	0,047	*	0,95	0,331	
GNG10	-0,30	0,280	-0,61	0,005	*	-0,10	0,752	
GPR137B	-0,51	0,376	-0,90	0,044	*	-0,31	0,566	
GPR35	-0,41	0,322	-0,70	0,031	*	-0,12	0,791	
GPR84	-0,38	0,154	-0,68	0,002	*	0,05	0,891	
GTF2I	0,85	0,101	1,11	0,009	*	0,75	0,082	
GZMM	0,55	0,215	0,77	0,029	*	0,31	0,448	
HBEGF	-0,57	0,080	-0,76	0,004	*	0,02	0,970	
HECTD3	0,61	0,107	0,66	0,038	*	0,38	0,267	
HLCS	0,46	0,310	0,80	0,024	*	0,59	0,105	
IFFO1	0,50	0,145	0,61	0,032	*	0,23	0,487	
IGSF6	-0,65	0,058	-0,85	0,003	*	-0,23	0,502	
IL19	-0,62	0,543	-1,55	0,033	*	-0,56	0,516	
IL1R2	-0,38	0,627	-1,20	0,025	*	-0,63	0,285	
IL24	-0,86	0,053	-0,92	0,013	*	-0,51	0,201	
IL6	-0,43	0,196	-0,68	0,012	*	0,14	0,701	
ILF3-AS1	-0,50	0,263	-0,76	0,035	*	-0,26	0,542	
ISY1-RAB43	0,18	0,841	-1,07	0,033	*	0,24	0,717	
JTB	0,24	0,621	0,73	0,030	*	0,33	0,372	
KCNA3	0,08	0,879	0,85	0,005	*	0,37	0,264	
KMO	-0,50	0,122	-0,72	0,006	*	-0,38	0,183	
LACC1	-0,40	0,071	-0,65	0,000	*	-0,27	0,171	
LILRB3	-0,31	0,410	-0,67	0,017	*	0,12	0,763	
MET	-0,36	0,419	-0,67	0,049	*	-0,06	0,916	
MMP1	-0,29	0,342	-0,74	0,001	*	-0,29	0,245	
MMP19	-0,34	0,254	-0,59	0,013	*	-0,32	0,204	
MTERFD1	-0,02	0,982	-0,61	0,048	*	-0,38	0,249	
NCF1	-0,48	0,214	-0,85	0,006	*	-0,39	0,241	
NCF1C	-0,56	0,254	-1,07	0,005	*	-0,53	0,198	
NENF	0,90	0,068	0,97	0,018	*	0,51	0,247	
OCEL1	0,70	0,198	1,04	0,018	*	0,57	0,232	
PEX26	0,54	0,179	0,83	0,010	*	0,47	0,166	
PGAM5	0,47	0,256	0,82	0,013	*	0,63	0,055	
PHF1	0,08	0,883	0,65	0,043	*	0,38	0,270	
PIGP	-0,03	0,970	-0,68	0,046	*	-0,24	0,557	
PILRA	-0,49	0,095	-0,75	0,002	*	-0,45	0,063	
PKIA	0,57	0,256	0,86	0,034	*	0,54	0,211	
PLA2G7	-0,53	0,058	-0,73	0,002	*	-0,33	0,187	
PLAUR	-0,32	0,229	-0,68	0,001	*	-0,21	0,363	
PMP22	-0,72	0,132	-1,08	0,005	*	-0,45	0,290	
PTAFR	-0,58	0,099	-0,73	0,012	*	0,08	0,867	
QPCT	-0,57	0,108	-0,81	0,005	*	-0,53	0,072	
RAB2A	0,45	0,198	0,60	0,033	*	0,53	0,059	
RAC1	0,90	0,065	1,13	0,005	*	0,76	0,063	
RALY	0,39	0,234	0,60	0,023	*	0,44	0,107	
RANBP10	0,14	0,738	0,59	0,026	*	0,31	0,293	
RASA3	0,38	0,120	0,66	0,001	*	0,34	0,102	
RAVER1	0,43	0,182	0,62	0,017	*	0,50	0,053	
RCN2	0,48	0,231	0,76	0,017	*	0,41	0,234	
RETSAT	0,48	0,231	0,65	0,047	*	0,59	0,065	
RSPH3	-0,13	0,841	-0,95	0,008	*	-0,29	0,497	
SEMA4A	-0,53	0,057	-0,69	0,003	*	-0,04	0,911	
SEPHS2	0,43	0,503	1,00	0,030	*	0,87	0,055	
SLAMF8	-0,27	0,544	-0,64	0,040	*	0,53	0,087	
SNAPC1	-0,34	0,325	-0,64	0,016	*	-0,30	0,300	
SUSD3	0,53	0,101	0,59	0,027	*	0,41	0,136	
TAF1A	-0,23	0,588	-0,60	0,043	*	-0,03	0,954	
TBC1D17	0,37	0,330	0,65	0,029	*	0,38	0,227	
TBC1D22A	0,38	0,254	0,62	0,020	*	0,23	0,469	
TBC1D25	0,37	0,298	0,71	0,010	*	0,44	0,120	
TBL2	0,31	0,450	0,68	0,026	*	0,23	0,542	
TFE3	0,23	0,552	0,61	0,027	*	0,41	0,159	
THBS1	-0,37	0,256	-0,64	0,012	*	-0,49	0,053	
TLR8	-0,74	0,089	-0,88	0,015	*	-0,03	0,963	
TMEM176B	-0,94	0,068	-1,03	0,016	*	-0,82	0,055	
TPRKB	-0,46	0,212	-0,88	0,003	*	-0,48	0,120	
UBE3A	0,06	0,924	0,65	0,042	*	0,54	0,096	
USP21	0,37	0,272	0,75	0,004	*	0,27	0,363	
VAMP4	-0,31	0,375	-0,61	0,024	*	-0,40	0,163	

VNN2	-0,47	0,086	-0,60	0,008	*	-0,41	0,074	
WBP5	-0,97	0,205	-1,42	0,020	*	-0,70	0,298	
XPOT	0,78	0,061	0,78	0,026	*	0,57	0,108	
ZDHHC3	0,56	0,095	0,73	0,008	*	0,30	0,328	
ZER1	0,58	0,161	0,78	0,019	*	0,36	0,333	
ZNF589	0,27	0,504	0,73	0,012	*	0,25	0,474	
ZNF71	0,55	0,101	0,65	0,020	*	0,23	0,487	
ZNF792	0,70	0,108	0,95	0,008	*	0,67	0,063	
ABHD14A	-0,24	0,527	0,00	1,000		-0,62	0,018	*
ACSL1	0,07	0,872	-0,08	0,823		0,62	0,014	*
AGO2	0,21	0,547	0,31	0,259		0,71	0,003	*
APOBEC3D	0,20	0,673	0,12	0,801		0,68	0,034	*
ARHGAP31	0,22	0,582	0,28	0,364		0,60	0,026	*
ATF3	0,18	0,771	0,04	0,949		0,89	0,017	*
B3GNT5	0,11	0,855	-0,01	0,985		0,81	0,013	*
BACH2	0,26	0,352	0,25	0,296		0,71	0,001	*
C19orf43	-0,57	0,055	-0,30	0,285		-0,59	0,014	*
C9orf69	0,34	0,319	0,40	0,153		0,64	0,012	*
CCL4	-0,15	0,757	-0,39	0,248		0,94	0,002	*
CCL8	-0,28	0,813	-0,94	0,214		2,02	0,003	*
CCR2	-0,56	0,174	-0,32	0,412		-1,00	0,002	*
CD160	-0,64	0,288	-0,74	0,143		2,02	0,000	*
CD274	-0,28	0,494	-0,36	0,285		0,78	0,008	*
CD36	-0,77	0,172	-0,72	0,129		-1,26	0,005	*
CD52	-0,08	0,769	-0,25	0,155		-0,61	0,000	*
CD69	0,02	0,974	-0,08	0,770		0,82	0,000	*
CD72	-0,30	0,499	-0,29	0,431		0,65	0,038	*
CHKA	-0,41	0,410	-0,56	0,156		-0,76	0,039	*
CLIC3	0,05	0,939	-0,05	0,913		-0,67	0,041	*
COMMD3-BMI1	0,34	0,655	0,21	0,771		-1,41	0,005	*
COX17	-0,21	0,616	-0,45	0,133		-0,67	0,015	*
CRTAM	-0,39	0,388	-0,39	0,310		1,05	0,002	*
CSF2	0,11	0,939	0,79	0,337		1,61	0,024	*
CSF2RB	0,04	0,930	0,01	0,989		0,73	0,004	*
CX3CR1	-0,19	0,662	-0,24	0,493		-0,64	0,023	*
CXCL10	-0,55	0,511	-0,36	0,648		1,58	0,007	*
CXCL9	-0,32	0,687	-0,05	0,956		1,61	0,002	*
DUSP1	0,85	0,133	0,82	0,088		0,93	0,043	*
DVL2	0,54	0,143	0,58	0,058		0,61	0,040	*
EGR2	-0,67	0,094	-0,45	0,210		0,85	0,008	*
EIF4EBP1	-0,46	0,260	-0,15	0,737		-0,81	0,010	*
EPSTI1	0,09	0,874	0,16	0,728		0,75	0,021	*
FAM91A1	0,35	0,338	0,50	0,083		0,59	0,034	*
FASLG	0,05	0,933	0,06	0,881		0,76	0,007	*
FBXO30	0,27	0,586	0,63	0,077		0,76	0,024	*
FGFBP2	-0,16	0,647	-0,23	0,384		-0,77	0,001	*
FUS	0,30	0,429	0,37	0,238		0,61	0,029	*
GBP1	-0,22	0,716	-0,34	0,475		1,07	0,005	*
GBP1P1	-0,19	0,820	-0,02	0,979		1,11	0,019	*
GBP2	0,04	0,924	0,02	0,956		0,61	0,004	*
GBP4	0,00	0,997	0,08	0,823		0,74	0,002	*
GBP5	0,00	1,000	0,06	0,921		1,14	0,002	*
GCHFR	-0,03	0,952	-0,16	0,574		-0,64	0,004	*
GIMAP8	0,37	0,298	0,49	0,092		0,59	0,035	*
GZMA	-0,28	0,298	-0,29	0,194		-0,61	0,003	*
GZMB	0,05	0,916	-0,08	0,829		0,71	0,002	*
GZMH	-0,23	0,512	-0,36	0,181		-0,68	0,005	*
HAVCR2	0,07	0,858	-0,19	0,435		0,66	0,001	*
HIST1H2BK	-0,29	0,391	-0,47	0,076		-0,71	0,005	*
HIVEP1	0,36	0,213	0,09	0,783		0,65	0,005	*
HSPBP1	0,53	0,153	0,49	0,120		0,78	0,007	*
HSPH1	0,40	0,414	0,71	0,056		0,72	0,046	*
IFNG	0,32	0,706	-0,32	0,654		1,21	0,024	*
IGF2R	0,15	0,664	0,37	0,119		0,66	0,003	*
IKZF2	0,20	0,664	0,42	0,212		0,61	0,049	*
IL21R-AS1	0,16	0,635	0,34	0,172		0,61	0,007	*
INADL	0,56	0,070	0,45	0,088		0,59	0,018	*
INHBA	-0,08	0,885	-0,24	0,549		0,89	0,005	*
INIP	-0,50	0,067	-0,38	0,111		-0,59	0,008	*
INTS3	0,55	0,092	0,49	0,079		0,67	0,010	*
IRF1	0,01	0,996	0,13	0,727		0,68	0,013	*
IRF4	0,36	0,256	0,41	0,113		1,03	0,000	*
IRF8	-0,09	0,790	0,01	0,982		0,71	0,000	*
IRG1	-0,36	0,567	-0,45	0,380		1,41	0,002	*
ISOC2	0,30	0,564	0,07	0,898		0,74	0,036	*
JAK2	0,63	0,087	0,53	0,091		0,98	0,001	*
JUN	0,06	0,880	0,23	0,400		0,76	0,002	*
KCNK6	0,37	0,503	0,60	0,144		0,80	0,040	*
KDM6B	-0,05	0,911	0,19	0,527		0,66	0,006	*
KHK	-0,81	0,070	-0,17	0,733		-0,73	0,047	*
KIR2DL4	-0,26	0,656	-0,39	0,402		1,02	0,008	*
KLRB1	-0,13	0,622	-0,26	0,178		-0,82	0,000	*
LGALS1	-0,42	0,178	-0,49	0,052		-0,69	0,005	*
LIMK2	-0,08	0,879	-0,01	0,976		0,79	0,006	*
LMNB1	0,13	0,793	0,30	0,418		0,72	0,018	*
LOC729013	-0,42	0,503	-0,39	0,469		-0,93	0,035	*
LRCH4	0,72	0,170	0,74	0,087		0,89	0,033	*
LST1	-0,45	0,432	-0,83	0,055		-1,03	0,013	*
MAP3K13	-0,09	0,837	-0,38	0,125		-0,64	0,006	*
MB21D1	0,00	1,000	0,15	0,701		0,76	0,009	*
MFGE8	0,08	0,941	0,21	0,792		-1,17	0,034	*
MPPE1	-0,31	0,477	-0,43	0,220		-0,77	0,013	*
NAB2	0,25	0,731	0,55	0,278		1,23	0,006	*
NDUFAF6	-0,27	0,557	-0,54	0,115		-0,65	0,044	*
NFKBIB	0,08	0,881	0,21	0,576		0,60	0,043	*
NOP14	0,14	0,821	0,55	0,164		0,80	0,028	*
NR4A1	0,12	0,777	0,06	0,871		0,95	0,000	*
NR4A2	-0,09	0,841	-0,25	0,373		0,75	0,002	*
OSBPL5	-0,55	0,152	-0,21	0,581		-0,62	0,046	*
PAGR1	0,48	0,159	0,54	0,051		0,64	0,016	*
PIM3	0,05	0,930	0,42	0,181		0,59	0,040	*
PLEK	-0,08	0,853	-0,17	0,579		0,82	0,001	*
PML	0,51	0,168	0,56	0,067		0,62	0,036	*
PPP1R15B	0,27	0,302	0,32	0,143		0,60	0,003	*
PTGES2	-0,39	0,545	-0,64	0,189		-1,00	0,026	*
PTGS2	0,12	0,854	-0,02	0,971		0,79	0,022	*
PYCARD	-0,16	0,703	-0,21	0,550		-0,60	0,032	*
RAB20	-0,28	0,509	-0,34	0,324		0,61	0,045	*
RGS16	-0,16	0,809	-0,07	0,901		1,09	0,004	*
RNF19A	0,22	0,457	0,26	0,282		0,67	0,002	*
RPL21	-0,54	0,132	-0,10	0,809		-0,84	0,004	*
RPL34	-0,07	0,837	-0,10	0,709		-0,59	0,003	*
RPS29	-0,02	0,975	-0,06	0,878		-0,60	0,024	*
SBNO2	0,14	0,758	0,23	0,515		0,59	0,044	*
SERPINE2	-0,19	0,655	0,02	0,957		0,63	0,026	*
SH2D1B	-0,11	0,749	-0,09	0,742		0,94	0,000	*
SH3BGRL3	-0,28	0,207	-0,26	0,180		-0,66	0,000	*
SLC25A20	-0,14	0,731	-0,33	0,257		-0,59	0,024	*
SLC7A5	0,33	0,355	0,21	0,530		0,60	0,024	*
SLMO2-ATP5E	-0,30	0,893	-0,82	0,594		-2,45	0,042	*
SMG6	0,30	0,410	0,45	0,121		0,62	0,021	*
SMIM11	-0,40	0,417	-0,43	0,299		-0,74	0,045	*
SMIM14	-0,32	0,165	-0,36	0,057		-0,68	0,000	*
SNHG8	-0,25	0,666	-0,37	0,400		-0,80	0,034	*
SPRY2	-0,16	0,880	-0,46	0,531		1,37	0,018	*
SRC	0,25	0,549	0,32	0,341		0,59	0,042	*
SREBF1	0,41	0,509	0,58	0,233		0,93	0,034	*
STARD4	0,21	0,583	0,33	0,268		0,83	0,002	*
STAT1	0,20	0,582	0,23	0,428		0,64	0,008	*
SUMF1	-0,17	0,683	-0,37	0,222		-0,64	0,017	*
TAGAP	0,03	0,937	0,08	0,793		1,06	0,000	*
TESK1	-0,06	0,921	0,18	0,645		0,63	0,038	*
TMEM165	0,42	0,303	0,57	0,083		0,65	0,038	*
TNF	-0,33	0,261	-0,32	0,206		0,61	0,007	*
TNFRSF10A	0,34	0,619	0,63	0,215		0,93	0,044	*
TNFRSF9	0,02	0,976	-0,09	0,807		1,08	0,000	*
TP53I13	0,53	0,210	0,62	0,073		0,84	0,010	*
TYSND1	-0,24	0,590	-0,10	0,818		-0,70	0,018	*
UBE2D1	0,63	0,111	0,62	0,066		0,91	0,005	*
XCL1	-0,07	0,941	0,02	0,979		2,19	0,000	*
XCL2	-0,25	0,805	0,77	0,257		2,83	0,000	*
ZNF326	-0,37	0,344	-0,53	0,088		-0,60	0,044	*

Supplementary Table 2: List of genes significantly (FDR 5%) and differentially (fold-change > 2) expressed after incubation of human PBMCs (n=7) with IL-7 compared to unstimulated cells.

Gene ID	logFC /Unstim	adj.P.Val
CISH	3,24	1,8E-16
SOCS2	2,91	7,8E-12
MEOX1	2,56	2,5E-07
DPP4	2,42	1,1E-14
BCL2	2,28	3,4E-10
TAF4B	2,27	9,9E-13
FRMD4B	1,96	5,4E-08
AFAP1	1,87	4,1E-08
PTGER2	1,85	1,5E-12
IGFBP3	1,84	9,3E-07
IRF4	1,82	5,6E-12
IFNG	1,66	1,4E-03
AHR	1,64	2,3E-07
DNLZ	1,63	1,2E-04
CMAHP	1,57	2,8E-08
ALDH16A1	1,54	2,5E-04
TXK	1,46	8,3E-08
ADAM19	1,46	5,8E-11
RGS1	1,44	4,3E-09
PSMD3	1,44	1,8E-04
CDK6	1,44	4,7E-07
PDE4B	1,43	1,2E-11
LTA	1,42	2,8E-06
MB21D2	1,39	1,2E-06
BHLHE40	1,39	2,5E-07
TNFRSF4	1,38	1,2E-03
SOS1	1,37	6,1E-09
ETS1	1,37	5,2E-05
FAM101B	1,36	4,7E-06
FAM13A	1,30	2,7E-04
TAF10	1,29	4,9E-03
CCR2	1,29	3,3E-05
AP3M2	1,28	3,1E-09
DDRGK1	1,28	7,0E-03
DOHH	1,26	4,9E-02
IKZF4	1,26	3,8E-05
SLC4A10	1,26	5,9E-05
KLC2	1,23	3,6E-03
COTL1	1,23	2,9E-04
CYLD	1,21	6,4E-13
SYNE3	1,19	9,6E-04
DUSP2	1,19	1,4E-02
CD8B	1,17	1,5E-09
ITGA4	1,16	1,2E-11
ST3GAL5	1,15	2,8E-02
IL2RA	1,15	1,8E-07
GNPDA1	1,15	2,8E-09
CCR5	1,15	2,1E-04
MAF1	1,14	2,0E-02
SLC37A3	1,14	1,7E-04
SPINT1	1,14	1,8E-02
FKBP5	1,14	2,7E-07
DDI2	1,13	2,9E-03
HSBP1L1	1,12	3,8E-03
MTA2	1,12	5,8E-04
UGCG	1,10	2,2E-03
TLR1	1,10	5,2E-05
C17orf59	1,07	1,4E-02
RNF149	1,07	5,3E-05
GNA15	1,07	7,9E-04
POLRMT	1,06	5,3E-05
HSPA1L	1,06	4,2E-03
CMTM6	1,04	7,3E-13
TRPC4AP	1,03	2,9E-04
PITRM1	1,03	2,5E-07
B3GNT2	1,03	1,1E-02
KLF7	1,02	5,7E-05
TAZ	1,01	3,2E-02

Gene ID	logFC /Unstim	adj.P.Val
NOG	-1,92	1,4E-03
MAP3K14-AS1	-1,74	1,4E-02
S100B	-1,61	1,6E-02
PASK	-1,55	1,9E-15
TSC22D3	-1,52	1,2E-15
TCF7	-1,43	3,2E-12
HPCAL4	-1,38	1,5E-04
GIMAP2	-1,34	1,1E-12
AVPI1	-1,32	2,7E-03
PF4	-1,32	2,3E-02
FAM102A	-1,13	2,1E-09
CBR3	-1,12	1,4E-03
GPA33	-1,11	1,4E-03
PHF19	-1,11	1,4E-03
ZCCHC18	-1,10	3,7E-02
IL7R	-1,10	1,4E-09
CD27	-1,09	1,4E-09
AQP3	-1,08	1,6E-04
SNHG7	-1,08	3,7E-02
LOC100507206	-1,07	4,7E-02
TMEM204	-1,06	3,4E-10
FAIM3	-1,03	4,4E-11
NCAPH	-1,02	3,4E-04
LY9	-1,01	6,6E-08
SELL	-1,00	6,4E-13
